# Characterization of the complete chloroplast genome of *Prunus davidiana*, an excellent horticultural species

**DOI:** 10.1080/23802359.2020.1719930

**Published:** 2020-01-29

**Authors:** Xi Wang, Junru Wang, Minrong Luo

**Affiliations:** a College of Life Sciences, Northwest A&F University, Yangling, Shaanxi, China;; b Herbarium, Northwest A&F University, Yangling, Shaanxi, China;; c College of Horticulture, Northwest A&F University, Yangling, Shaanxi, China;; d Research Center of Horticulture Science, Northwest A&F University, Yangling, Shaanxi, China

**Keywords:** Chloroplast genome, *Prunus davidiana*, phylogenetic analysis

## Abstract

*Prunus davidiana* (Rosaceae) is of great importance horticulturally as the rootstock of some fruit trees. Here, the complete chloroplast genome of *P. davidiana* was assembled based on the Illumina reads. The complete cp genome of *P. davidiana* was 157,660 bp in length and contained a pair of inverted repeat (IR, 26,387 bp) regions, which were separated by the small single-copy (SSC, 19,122 bp) and the large single-copy (LSC, 85,764 bp) regions. It encoded 135 genes. The phylogenetic analysis revealed that *P. davidiana* is sister to other peaches. This result will be useful for future studies of the lineage.

*Prunus davidiana* (Carriére) de Vos ex L. Henry (Rosaceae) is always used as the rootstock of fruit trees such as peach, plum in China, and also as an ornamental plant with attractive pink flowers, showing a great horticultural value (Lu 1986). Here, the chloroplast (cp) genome of *P. davidiana* was assembled by using the genome skimming approach (Zimmer and Wen 2015). It will be useful for future studies on the taxonomy and phylogeny of the economically important peach lineage of *Prunus* (Zhao et al. 2016).

*Prunus davidiana* was sampled from Mt. Taibaishan, Meixian County, China (107°40′34′′E, 34°02′33′′N, alt. 945 m). A voucher specimen (LMR20160501) was deposited in the Herbarium of Northwest A&F University (WUK), China. Total genomic DNA was extracted with the modified cetyltrimethylammonium bromide (CTAB) method (Doyle and Doyle 1987). The extracted DNA was sequenced using the Illumina Miseq platform (Illumina, San Diego, CA). Reads of the cp genome were assembled using GetOrganelle (Jin et al. 2018). The annotation of the cp genome sequence was performed using PGA (https://github.com/quxiaojian/PGA). The genome map was generated in the webserver OGDRAW (http://ogdraw.mpimp-golm.mpg.de) (Lohse et al. 2013). The annotated complete cp genome sequence was submitted to GenBank (Accession Number MK634746).

The complete cp genome of *P. davidiana* was 157,660 bp in length and contained a pair of inverted repeat (IR, 26,387 bp) regions, which were separated by a small single-copy (SSC, 19,122 bp) region and a large single-copy (LSC, 85,764 bp) region. The whole cp genome encoded 135 genes including 88 protein-coding genes (PCG), 39 tRNA genes, 8 rRNA genes. Of these genes, 16 genes (*atpF*, *ndhA*, *ndhB*, *petB*, *petD*, *rpl2*, *rpl16*, *rpoC1*, *rps12*, *rps16*, *trnA-UGC*, *trnI-GAU*, *trnG-UCC*, *trnK-UUU*, *trnL-UAA*, and *trnV-UAC*) had one intron and 2 genes (*clpP* and *ycf3*) had two introns. Most genes occurred in a single copy, while six PCGs (*ndhB*, *rpl2*, *rpl23*, *rps7*, *rps12*, and *ycf2*), seven tRNA genes (*trnA-UGC*, *trnI-CAU*, *trnI-GAU*, *trnL-CAA*, *trnN-GUU*, *trnR-ACG*, and *trnV-GAC*), and four rRNA genes (*rrn4.5*, *rrn5*, *rrn16*, and *rrn23*) in IR regions are duplicated. The overall GC content of *P. davidiana* cp genome is 36.8% with 34.6, 30.3, and 42.6% GC in the LSC, SSC, and IR regions, respectively.

Totally 12 additional cp genomes of the genus *Prunus* L. were used to obtain the phylogenetic position of *P. davidiana*. *Prinsepia utilis* and *Prinsepia sinensis* were used as the outgroup. All the cp genome sequences were aligned in MAFFT (Katoh and Standley 2013), which was implemented in Geneious R10 (Biomatters Ltd., Auckland, New Zealand). We reconstructed a phylogeny under the maximum-likelihood criterion in RAxML (Stamatakis 2014) and determined the clade support using 10,000 bootstrap replicates. The phylogenetic tree shows that *P. davidiana* and other species of subgenus *Amygdalus* form a small clade (Figure 1), which is consistent with the prior phylogenetic studies of *Prunus* based on several molecular markers (Bortiri et al. 2001; Wen et al. 2008; Zhao et al. 2016). We expect that the cp genome of *P. davidiana* will be valuable for future horticultural studies.

**Figure 1.**
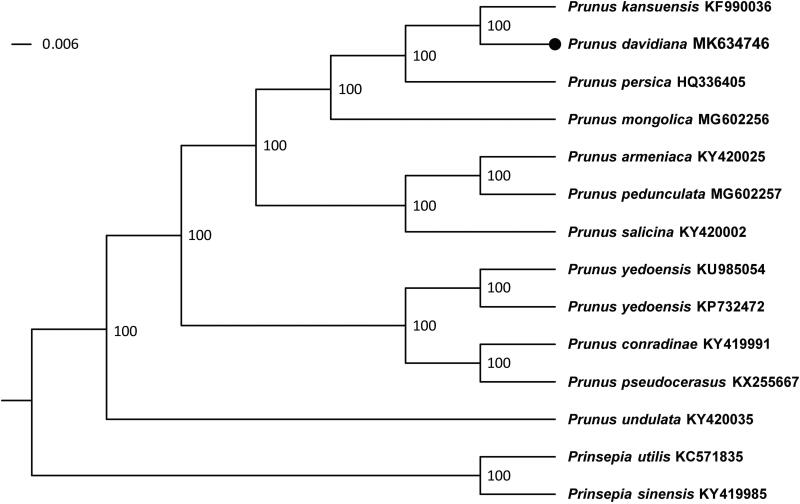
Maximum-likelihood phylogenetic tree for genus *Prunus* L. based on 14 complete chloroplast genomes. The number on each node indicates bootstrap support value >50% for each clade. Accession Numbers: *Prunus kansuensis* (KF990036), *Prunus davidiana* (MK634746), *P. persica* (HQ336405), *Prunus mongolica* (MG602256), *Prunus armeniaca* (KY420025), *Prunus pedunculata* (MG602257), *Prunus salicina* (KY420002), *Prunus yedoensis* (KU985054), *Prunus conradinae* (KY419991), *Prunus pseudocerasus* (KX255667), *Prunus undulata* (KY420035), *Prinsepia utilis* (KC571835), *Prinsepia sinensis* (KY419985).
